# Retrospective financial analysis of medicines reimbursement services in community pharmacy

**DOI:** 10.1186/2052-3211-8-S1-P6

**Published:** 2015-10-05

**Authors:** Elena Chitan

**Affiliations:** 1 Department of Social Pharmacy ‘V. Procopisin’, State University of Medicine and Pharmacy ‘Nicolae Testemitanu’, Chisinau, MD 2025, Republic of Moldova

## Background

A problem for middle-income countries is unequal access to medicines. In Republic of Moldova (RM), reimbursed medicines did not decrease the burden of expenditures for medicines. In this context, the aim of this study was: evaluation of financial attractiveness for community pharmacies to provide medicine reimbursement services and factors that influence this process.

## Methods

As methods of study we used a survey, direct observation, break-even analysis[1] and literature review. The research was started in April 2014 and ended in May 2015. The study addressed revision of mark-up applied by wholesale and retail companies for reimbursed medicines, to improve availability and affordability for the out-patient sector on reimbursed medicines[3] and to analyze the capacity of pharmacists in providing cognitive services for reimbursed medicines, with the future possibility of pay for performance service implementation from the National Health Insurance Company (NHIC)[2,4]. To assess the cognitive implication level and the working time spent by pharmacists for dispensing one reimbursed drug, one unreimbursed Rx drug and one OTC drug, we asked pharmacists' opinions working in community pharmacies through a sociological survey, in a sample of 300 people. Simultaneously, time was assessed by direct observation in the pharmacy. The financial analysis was made using data collected from the National Bureau of Statistics and NHIC, for 314 pharmacies in 2014.

## Results

**Figure 1:**
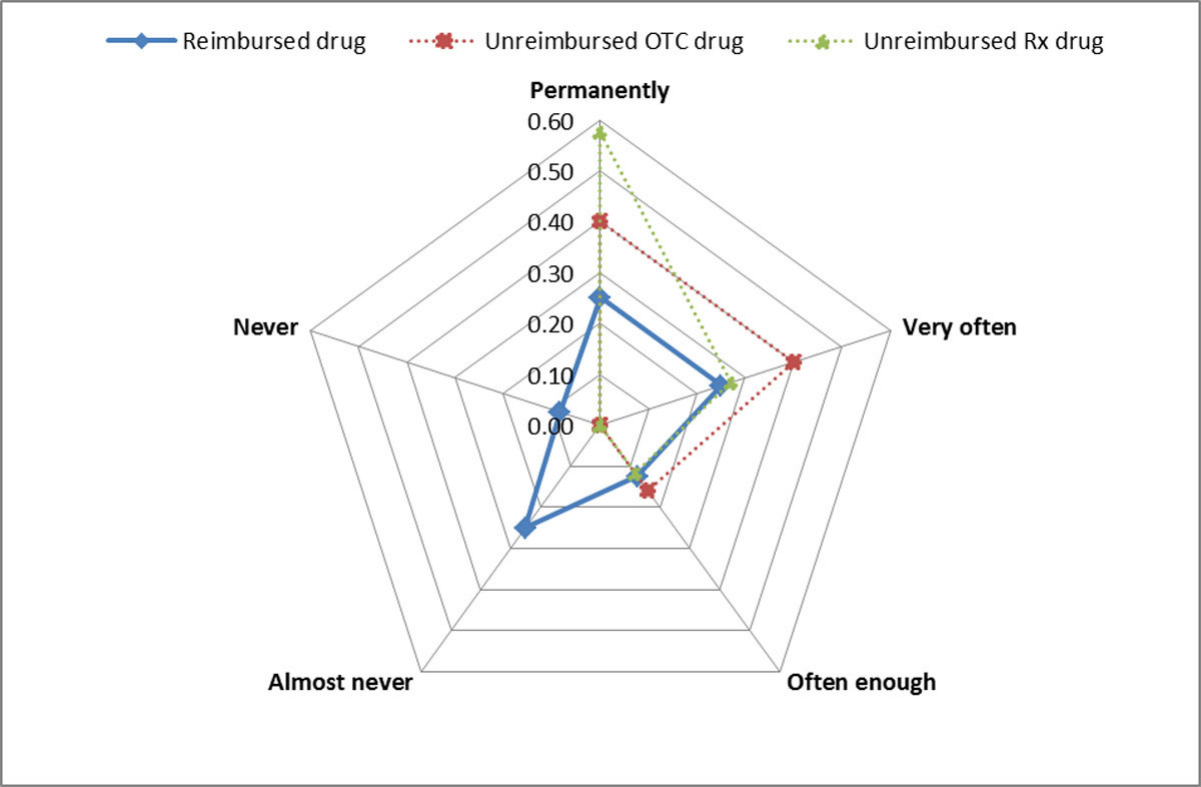
Relative frequency of cognitive implication

**Figure 2:**
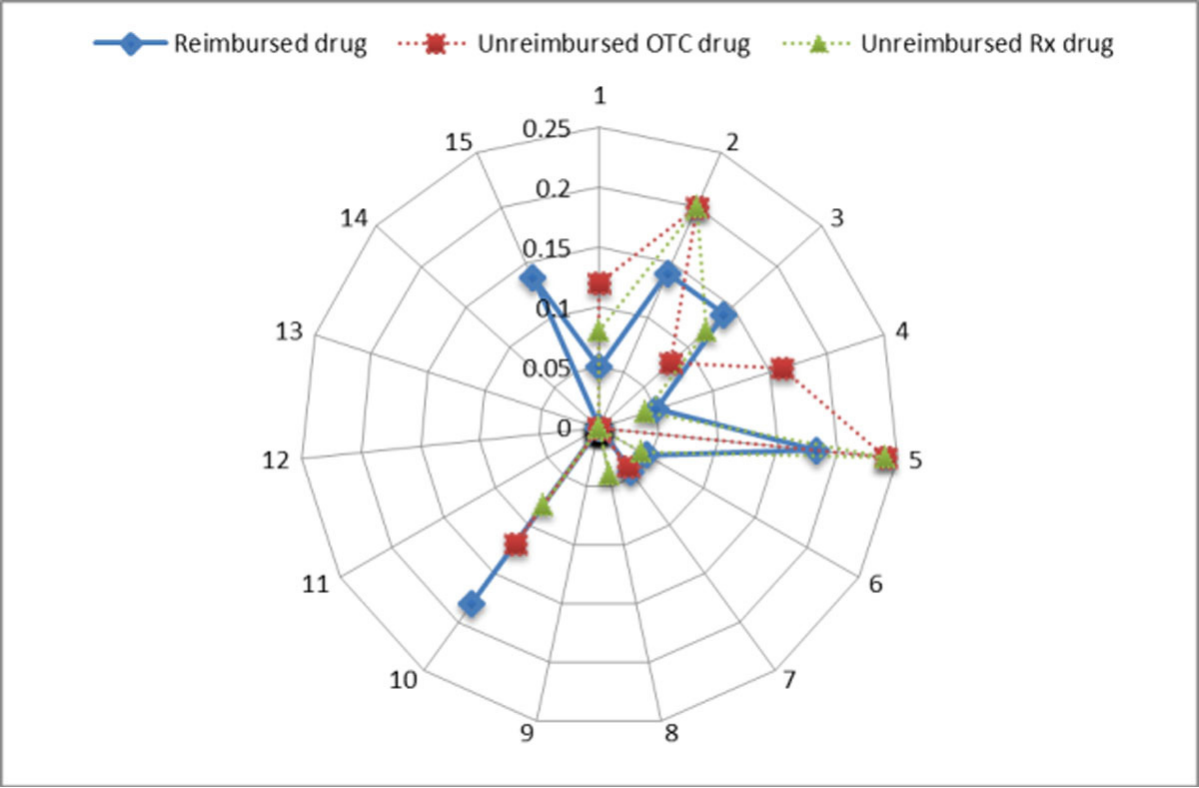
Relative frequency of dispensing time

**Figure 3:**
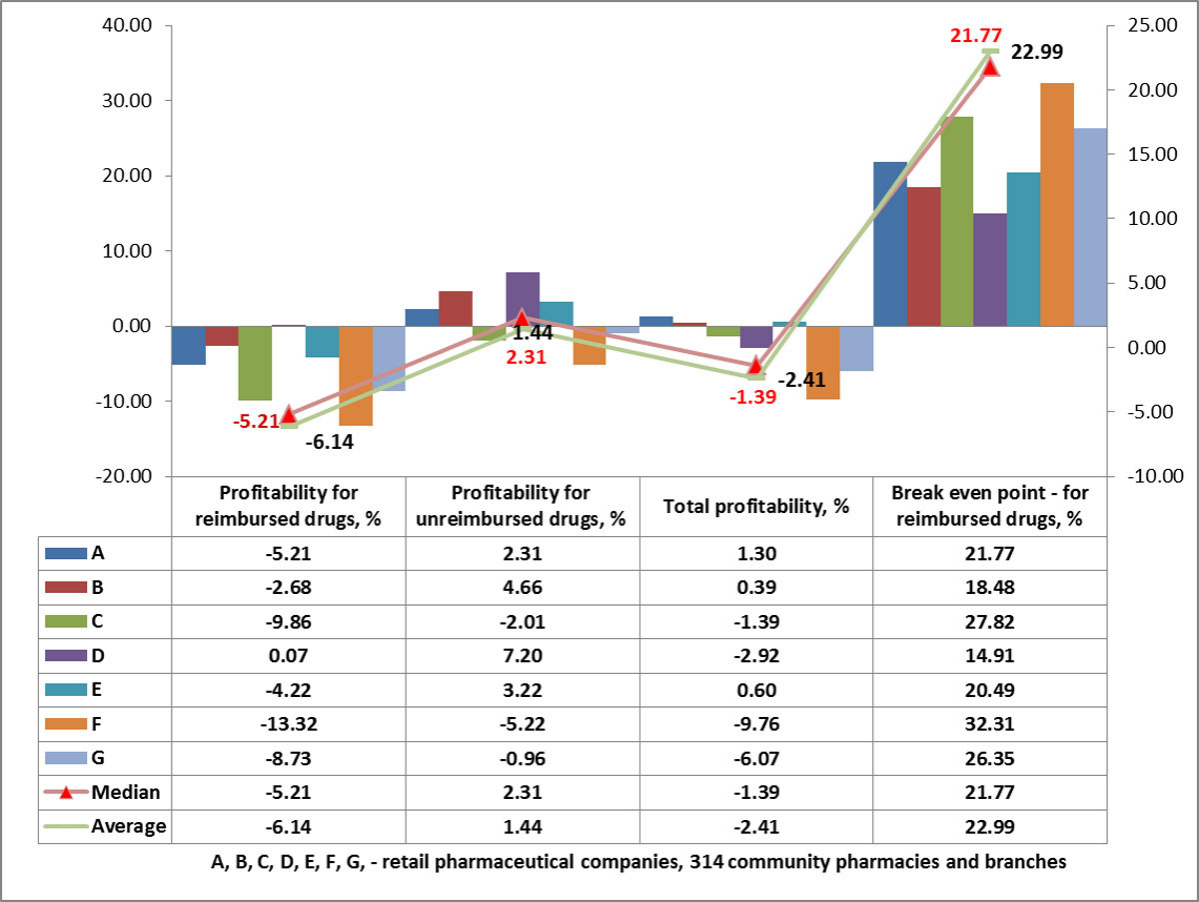
Financial analysis of pharmaceutical retail enterprises in RM, 2014

The cognitive implication for reimbursed drugs was ‘almost never’ or ‘permanently’, with a relative frequency of 0.25 (Figure 1), and the most common time spent by pharmacists for dispensing was 10 minutes, with a relative frequency of 0.18 (Figure 2). These results confirm that the pharmacist loses most of their time for technical processing of the prescriptions, a fact demonstrated through direct observation of the process. The median of profitability for reimbursed drugs was -5.21%, for unreimbursed drugs +2.16% (Figure 3). The break-even point for reimbursed drugs is 22% of mark-up. The results show that it is not convenient for pharmacies to dispense medicines with reimbursed prescription because of a lack of benefit for them.

## Conclusions

It is recommended to review the added mark-ups for reimbursed medicines in the retail pharmaceutical sector from 15% to 22% (using regressive mark-ups); and to introduce continuing education courses for pharmacists in the field of adherence and compliance to treatment for patients with non-communicable diseases, for medicines from the reimbursement list.
